# Delay in the diagnosis of *Brucella abortus* bacteremia in a nonendemic country: a case report

**DOI:** 10.1186/s12879-024-09377-y

**Published:** 2024-05-13

**Authors:** Jae Hyeon Park, Taek Soo Kim, Hyunwoong Park, Chang Kyung Kang

**Affiliations:** 1 Department of Laboratory Medicine, Seoul National University Hospital, Seoul, Republic of Korea; 2 Department of Laboratory Medicine, Seoul National University College of Medicine, Seoul, Republic of Korea; 3 Department of Laboratory Medicine, Seoul National University Boramae Medical Center, Seoul, Republic of Korea; 4 Department of Internal Medicine, Seoul National University College of Medicine, 101 Daehak-Ro, Jongno-Gu, Seoul, 03080 Republic of Korea

**Keywords:** *Brucella abortus*, Brucellosis, Bacteremia, MALDI–TOF MS, Whole-genome sequencing

## Abstract

**Background:**

It is challenging to diagnose brucellosis in nonendemic regions because it is a nonspecific febrile disease. The accurate identification of *Brucella* spp. in clinical microbiology laboratories (CMLs) continues to pose difficulties. Most reports of misidentification are for *B. melitensis*, and we report a rare case of misidentified *B. abortus*.

**Case presentation:**

A 67-year-old man visited an outpatient clinic complaining of fatigue, fever, and weight loss. The patient had a history of slaughtering cows with brucellosis one year prior, and his *Brucella* antibody tests were negative twice. After blood culture, the administration of doxycycline and rifampin was initiated. The patient was hospitalized due to a positive blood culture. Gram-negative coccobacilli were detected in aerobic blood culture bottles, but the CML's lack of experience with *Brucella* prevented appropriate further testing. Inaccurate identification results were obtained for a GN ID card of VITEK 2 (bioMérieux, USA) and matrix-assisted laser desorption ionization–time of flight mass spectrometry (MALDI–TOF MS) using a MALDI Biotyper (Bruker, Germany). The strain showed 100.0% identity with *Brucella* spp. according to 16S rRNA sequencing. MALDI–TOF MS peaks were reanalyzed using the CDC MicrobeNet database to determine *Brucella* spp. (score value: 2.023). The patient was discharged after nine days of hospitalization and improved after maintaining only doxycycline for six weeks. The isolate was also identified as *Brucella abortus* by genomic evidence.

**Conclusion:**

Automated identification instruments and MALDI–TOF MS are widely used to identify bacteria in CMLs, but there are limitations in accurately identifying *Brucella* spp. It is important for CMLs to be aware of the possibility of brucellosis through communication with clinicians. Performing an analysis with an additional well-curated MALDI–TOF MS database such as Bruker security-relevant (SR) database or CDC MicrobeNet database is helpful for quickly identifying the genus *Brucella*.

## Introduction

Diagnosing brucellosis in nonendemic regions is challenging because it is a nonspecific febrile disease, and appropriate testing is critical [1, 2]. Although serologic tests and PCR can be used to diagnose brucellosis, the standard test method is to detect *Brucella* spp. via blood culture. However, it remains challenging to accurately identify *Brucella* spp. in clinical microbiology laboratories (CMLs), especially in nonendemic countries. *Brucella*, classified as a Category B biological warfare pathogen by the Centers for Disease Control and Prevention (CDC), illustrates the importance of proper suspicion and testing protocols to avoid diagnostic delays and potential laboratory-acquired infections (LAIs) [2]. Laboratories lacking experience in *Brucella* diagnosis may encounter misidentification, particularly with automated identification instruments. While human brucellosis is predominantly caused by *Brucella melitensis*, *Brucella abortus*, and *Brucella suis* [1], most misidentification reports are limited to *B. melitensis* and *B. suis* [3–11]. We present a case involving the delayed diagnosis of *B. abortus* bacteremia, which was initially misidentified, and a review of the relevant literature on the misidentification of brucellosis, laboratory safety, and nomenclature issues.

## Case presentation

### Patient’s initial history

A 67-year-old man presented with weight loss of 7 kg over two months, fatigue, and fever. The patient is a livestock farmer whose cattle were diagnosed with brucellosis 14 months prior and culled and had since undergone two brucellosis microagglutination tests (MATs), both of which were non-reactive. The patient underwent oropharyngectomy for tonsil cancer three years prior and was on medication for hypertension, diabetes, and dyslipidemia. An outpatient blood test revealed a hemoglobin level of 11.4 g/dL, a white blood cell count of 4.7 × 10^9^/L (neutrophil percentage 92%), a platelet count of 113 × 10^6^/L, and an elevated C-reactive protein (CRP) level of 32.7 mg/L. The patient was seen in an infectious disease outpatient clinic the following week, where blood cultures and *Brucella* antibody tests were performed; doxycycline and rifampin were prescribed according to World Health Organization guidelines [12]. Two pairs of blood drawn from peripheral veins were inoculated into BACT/ALERT FA Plus and BACT/ALERT FN Plus (bioMérieux, Durham, NC, USA) and incubated in the BACT/ALERT Virtuo system (bioMérieux). Two aerobic bottles were positive after 48 and 62 h, respectively, and small Gram-negative coccobacilli were identified in the positive blood culture media. The patient was admitted for antibiotic treatment for suspected brucellosis two days after a positive blood culture and was admitted to an isolation unit because he had been diagnosed with coronavirus disease 2019 (COVID-19) two days earlier. The isolated Gram-negative coccobacilli formed small gray colonies after 48 h of incubation on sheep blood agar plate (Asan Pharmaceutical, Seoul, Republic of Korea) at 35 °C and 5% CO_2_ and did not grow on MacConkey agar plate (Asan Pharmaceutical). Due to a lack of experience in diagnosing brucellosis, the laboratory omitted essential biochemical tests, such as oxidase, catalase, and urease tests, all of which should yield positive results in the isolate. The isolate was identified as *P. fluorescens* by a GN ID card with the VITEK 2 system (bioMérieux) but failed to be identified using the Phoenix M50 (Becton Dickson, Franklin Lakes, NJ, USA). Gentamicin and piperacillin/tazobactam were added based on the identification results.

### MALDI–TOF MS and 16S rRNA sequencing

On the third day of hospitalization, the infectious disease physician requested accurate identification due to discrepancies in the clinical presentation and pathogen identification. Matrix-assisted laser desorption ionization–time-of-flight mass spectrometry (MALDI–TOF MS) was performed using a microflex LT (Bruker Daltonics, Bremen, Germany), and the results were analyzed with the MALDI Compass Library (DB9607, version 10.0) based on MALDI Biotyper Compass software 4.1. MALDI–TOF MS showed an unreliable identification as *Ochrobactrum grignonense* (score value: 1.411). The isolate was 16S rRNA sequenced using universal primers, DNA amplified with 27F/1492R primers, and sequenced with 785F/907R primers. Sequences were retrieved from the GenBank database using the BLAST algorithm and interpreted according to CLSI guidelines [13]. 16S rRNA sequence analysis of the isolate showed 100% identity to several species of the genus *Brucella*. The patient was released from isolation on Day 6 because he did not develop COVID-19-related pulmonary infiltrates, symptoms, or desaturation. On Day 7, the national reference laboratory reported a brucellosis MAT result of 1:320, strongly suggesting brucellosis. Peaks from the MALDI-TOF MS were reanalyzed and the Centers for Disease Control and Prevention (CDC) MicrobeNet’s MICROBENET 2022 1.0 library (https://microbenet.cdc.gov) yielded results for *Brucella* sp (score value: 2.023). After consulting with Bruker Korea and using a Security-Relevant (SR) database to reanalyze the peaks, the isolate was identified as *B. melitensis* (score value: 2.29).

### Patient treatment course

At the time of hospitalization, the patient complained of back pain and underwent lumbosacral spine magnetic resonance imaging, but there was no evidence of infectious spondylitis. He also underwent endoscopy, transthoracic echocardiography, and transesophageal echocardiography, which were unremarkable. A follow-up blood culture performed on hospital Day 1 was negative, and he was discharged on hospital Day 9 with resolution of fever. After discharge, doxycycline was maintained for a total of 6 weeks. Three weeks after discharge, his CRP level decreased to 0.9 mg/L, and he had regained weight three months later.

### Whole-genome sequencing

**Fig. 1:**
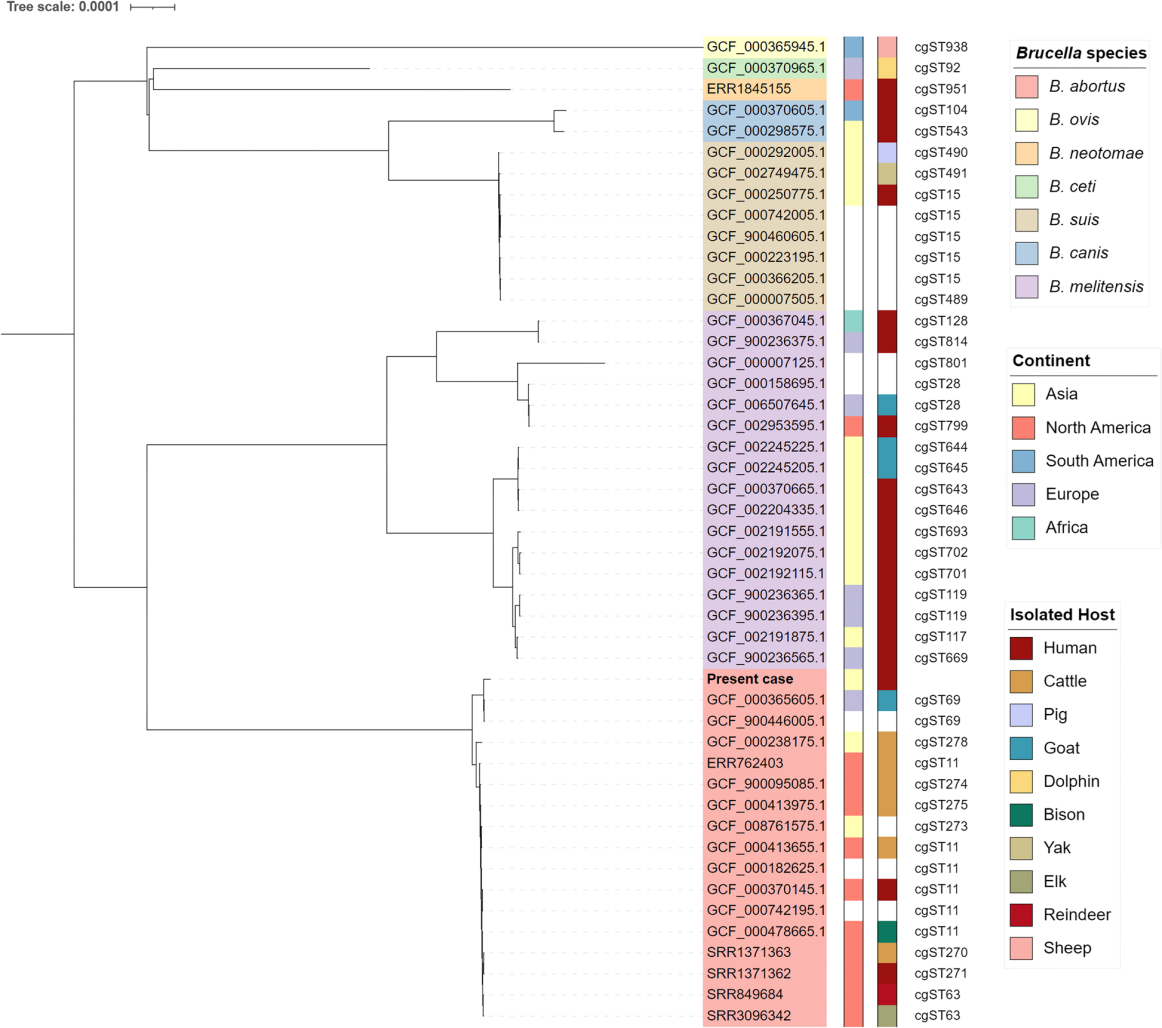
Maximum-likelihood tree based on representative *Brucella* genomes using a core genome multilocus typing scheme. This clinical isolate clustered with *B. abortus* and was found to be close to *B. abortus* cgST69, which was isolated from a goat in Europe. Abbreviations: cgST, core genome multilocus typing sequence type

Colonies were subjected to heat inactivation at 95 °C for 20 min, followed by DNA extraction with the MagNA Pure 96 system (Roche Diagnostics, Mannheim, Germany) and whole-genome sequencing (WGS) using the MiSeq platform (Illumina, San Diego, CA) through the MAFGEN project (CJ Bioscience, Suwon-si, Republic of Korea), as previously described [14]. After quality control with Trimmomatic [15], the reads were assembled with Unicycler v.0.5.0 [16]. The assembled genome was 3,186,837 bp, with a GC content of 57.8%, 73 contigs, an N50 of 109,700 bp and a complete BUSCO of 98.4%, indicating good assembly quality [17, 18]. The genome was analyzed using the EzBioCloud Genome Database (CJ Bioscience), which showed an absolute nucleotide identity of 99.98% with *B. abortus,* followed by 99.73% with *Brucella microti* [19]. Core genome multilocus typing (cgMLST) with 1,764 genes using representative strains of *Brucella* spp. showed that this clinical strain clustered with *B. abortus* [20] (Fig. 1). It was found to be close to *B. abortus* cgMLST sequence type 69 but was somewhat different, with 22 allele differences. In addition, it shows 93 allele distances with the only *B. abortus* strain reported in South Korea.

## Discussion and conclusions

Diagnosing brucellosis, especially in nonendemic areas or from returning travelers, is challenging [1, 2]. In this case, insufficient experience in conducting appropriate biochemical testing delayed accurate diagnosis, thereby increasing the risk of LAI [21]. However, brucellosis was subsequently confirmed during hospitalization through additional MALDI–TOF MS database and 16S rRNA sequencing. In South Korea, *B. abortus* is the main pathogen of human and bovine brucellosis, and brucellosis was designated a notifiable infectious disease in 2000. The first human case was reported in 2002, with reports increasing to more than 250 cases in 2006 [22]. Since then, human brucellosis incidence has been on the decline due to active eradication policies, with fewer than 10 cases per year since 2014 [23]. However, imported cases of *B. melitensis* have been reported [24, 25], requiring vigilance by CMLs.

Reports on the misidentification of *Brucella* spp. were most often with *B. melitensis* or *B. suis* being misidentified as *Ochrobactrum anthropi* (Table 1). Misidentification not only delays correct diagnosis but also increases the risk of LAI [2]. Manipulating unknown *Brucella* isolates on an open bench rather than in a biosafety cabinet (BSC) exposes many workers through aerosolization and increases the risk of LAI. In a recent assessment of the risk of exposure to brucellosis in laboratory workers in New York from 2015 to 2017, *Brucella* exposure incidents occurred in 10 of 11 confirmed brucellosis cases [26]. In the present case, brucellosis was clinically suspected, and the worker wore a mask and conducted all work in a Class II BSC, so there was no exposure. The worker was monitored for fever but did not develop symptoms. The use of MALDI–TOF MS is increasing, and safe work practices, including working with slow-growing organisms in a BSC and not using MALDI–TOF MS unless a biothreat agent is excluded, are recommended [26].

**Table 1:** Misidentified cases related to the genus *Brucella*

Case	Initial ID method	Initial ID	Correct ID	Brucella antibody
Elsaghir et al., 2003 [3]	API 20NE	*Ochrobactrum anthropi*	*Brucella melitensis*	Positive
Horvat et al., 2011 [4]	RapID NF Plus	*O. anthropi*	*Brucella suis*	NT
Carrington et al., 2012 [5]	RapID NF Plus	*O. anthropi*	*B. suis*	Positive
Vila et al., 2016 [6]	VITEK 2	*O. anthropi*	*B. suis*	Positive
Trêpa et al., 2018 [8]	VITEK MS	*O. anthropi*	*B. melitensis*	NT
Poonawala et al., 2018 [7]	VITEK MS	*O. anthropi*	*B. melitensis*	NT
Khaliulina Ushakova et al., 2020 [9]	Bruker MALDI Biotyper	*O. anthropi*	*B. melitensis*	Positive
Mori et al., 2020 [27]	VITEK 2	*B. melitensis*	*Haematobacter massiliensis*	Negative
	VITEK 2	*B. melitensis*	*Herbaspirillum frisingense*	NT
Gopalsamy et al., 2021 [10]	VITEK 2	*O. anthropi*	*B. suis*	Positive
Inal et al., 2022 [11]	Bruker MALDI Biotyper	*Ochrobactrum deajoenense*	*B. melitensis*	Positive
Current case	VITEK 2	*Pseudomonas fluorescens*	*Brucella abortus*	Positive

*Abbreviations: ID* identification, *NT* not tested

MALDI–TOF MS is commonly used for rapid and accurate identification of microorganisms. The Bruker SR database has been reported to be able to rapidly and accurately identify biothreat agents, including *Brucella* spp., while the in vitro diagnostics (IVD) and research use only (RUO) databases cannot [28]. In the identification of *Brucella* using VITEK MS, the IVD database failed, but the RUO database was reported to identify 56.9% of strains at the genus level [28]. In this case, the CDC MicrobeNet database identified the isolate as *Brucella* spp. and the Bruker SR database identified it as *B. melitensis*, both of which were successful in differentiating the genus *Brucella*. However, due to export restrictions, SR databases are not readily available for CMLs, especially those outside of Europe [29]. The use of MALDI-TOF MS to differentiate *Brucella* is not limited to the development of in-house databases; it also extends to reports that have been integrated into primary or public databases [30, 31]. Therefore, if MALDI–TOF MS is performed in a situation where brucellosis is suspected, it would be helpful to use publicly available CDC MicrobeNet database.

The nomenclature of the genus *Brucella* has long been controversial [32]. Recently, a reclassification of *Ochrobactrum* spp. to the genus *Brucella* was proposed due to genomic similarities [33], and both classifications are currently "validly published" nomenclature [34]. As the new classification has been applied to some microbial identification systems, guidelines have been published to reduce clinical confusion [35–37]. Given the known limitations of automated identification methods, including MALDI–TOF MS, in differentiating *Brucella* spp. and *Ochrobactrum* spp., it is important to distinguish them by morphologic and phenotypic characteristics [36]. This clinical isolate did not grow on MacConkey agar, suggesting *Brucella* spp.

Traditionally, subtyping of *Brucella* spp. has been based on multilocus variable-number tandem-repeat analysis (MLVA) [38]. With the increase in the amount of available WGS data, cgMLST for *B. melitensis*, which can be used to accurately perform epidemiological studies and outbreak analyses, has been developed [38]. In this study, a new *Brucella*-wide cgMLST scheme was used to perform phylogenetic analysis [20]. This clinical isolate is different from a previously reported *B. abortus* strain in South Korea. Although there is a lack of WGS data on *B. abortus* strains in South Korea, cgMLST may allow for more accurate analysis of transmission.

The limitations of automated identification systems for identifying *Brucella* spp. are well-recognized. Although MALDI–TOF MS is widely used in CMLs, it has limitations in identifying *Brucella* spp. without additional analysis. CMLs in nonendemic areas also require attention regarding the diagnosis of brucellosis because of diagnostic delays and the risk of LAI. It is important that clinicians' suspicions are well communicated and that CMLs perform appropriate testing with precaution to biosafety.

## Data Availability

This Whole Genome Shotgun project has been deposited at GenBank under the accession JARUPL000000000.

## Abbreviations

BSC: Biosafety cabinet
CML: Clinical microbiology laboratory
cgMLST: Core genome multilocus typing
COVID-19: Coronavirus disease 2019
CRP: C-reactive protein
LAI: Laboratory-acquired infection
MALDI–TOF MS: Matrix-assisted laser desorption ionization–time of flight mass spectrometry
IVD: In vitro diagnostics
RUO: Research use only
SR: Security-relevant
WGS: Whole-genome sequencing
